# Death-Associated Protein Kinase 1 (DAPK1) Protects against Myocardial Injury Induced by Myocardial Infarction in Rats via Inhibition of Inflammation and Oxidative Stress

**DOI:** 10.1155/2022/9651092

**Published:** 2022-01-17

**Authors:** Jun Zhang, Jing Zhang, Bo Zhou, Xiaojing Jiang, Yanrong Tang, Zhenzhen Zhang

**Affiliations:** Department of Cardiology, Chengdu First People's Hospital, Chengdu, China

## Abstract

**Objective:**

Heart failure and ventricular remodeling after acute myocardial infarction (AMI) are important factors affecting the prognosis of patients. Therefore, we expected to explore the therapeutic target of AMI by studying the effect of death-associated protein kinase 1 (DAPK1) on AMI rat model.

**Materials and Methods:**

We used male Sprague-Dawley rats to make AMI model, and after 1, 3, 7, and 14 d, we detect the success rate of modeling and the expression change of DAPK1 through 2, 3, 5-triphenyl tetrazolium chloride staining, myocardial injury markers detection, echocardiographic detection, and histological experiment. In addition, we determined the effect of DAPK1 on AMI by subcutaneous injection of the DAPK1 inhibitor (TC-DAPK 6). The effect of DAPK1 on cardiomyocytes has also been verified in cell experiments on H9c2 cells.

**Results:**

The expression of DAPK1 in AMI rats was significantly higher than that in sham group, and it increased with time. The expression of inflammatory factors (interleukin- (IL-) 1*β*, IL-6, and tumor necrosis factor-*α*) in AMI rats treated by TC-DAPK 6 was reduced. In addition, TC-DAPK 6 also reduced the activity of malonaldehyde and increased the activities of superoxide dismutase, glutathione, and catalase. The expression of antioxidant molecules such as peroxiredoxin1/4 and glutathione peroxidase1/3 was also promoted by TC-DAPK 6. In H9c2 cells, TC-DAPK 6 also reduced its oxidative stress level.

**Conclusions:**

The increase of DAPK1 may be related to the pathogenesis of AMI. DAPK1 inhibitors protect cardiomyocytes from AMI-induced myocardial injury by reducing levels of inflammation and oxidative stress in myocardial tissue and cells.

## 1. Introduction

Acute myocardial infarction (AMI) is a cardiovascular disease with high morbidity and high mortality [1]. Current treatment methods include drug thrombolysis, coronary intervention, and coronary artery bypass grafting. Although these treatments have benefited more and more patients, and the mortality rate has decreased significantly, the heart cannot be repaired by the regeneration of myocardial cells due to the poor regeneration of myocardial cells after infarction [2]. The loss of myocardial cells after AMI and the subsequent ventricular remodeling are the main causes of a series of complications after AMI, especially heart failure, which affects the prognosis of patients [3]. How to promote the repair and functional reconstruction of myocardial cells in the necrotic area after AMI and prevent ventricular remodeling has become the key to improving the prognosis of MI patients.

Death-associated protein kinase 1 (DAPK1) was discovered by Deiss in 1995 using gene cloning analysis technology in HeLa cell apoptosis induced by interferon-*γ* [4]. Recent studies have found that DAPK1 is an important serine/threonine kinase and is involved in a variety of cell-to-cell interactions, such as apoptosis, autophagy, cellular blebbing, tumor metastasis, and inflammation [5, 6]. A study found that the inhibition of DAPK1 can reduce the inflammatory factors in lung tissue and inhibit the level of oxidative stress by regulating the activity of p38MAPK/NF-*κ*B signaling pathway, thereby alleviating lung injury [7]. In addition, DAPK1 was also found to be regulated by resveratrol and affect autophagy in human skin fibroblasts [8]. DAPK1 has also been found to affect apoptosis and autophagy in various tumor cells [6]. In the process of myocardial infarction, myocardial cell ischemia and hypoxia lead to myocardial cell necrosis and apoptosis. In addition, local inflammation and oxidative damage are also important reasons for aggravating myocardial damage [2]. In view of the above-mentioned physiological functions of DAPK1, we speculated that DAPK1 may play a role in AMI. However, there are few studies on the effect of DAPK1 on AMI. Therefore, in this study, we made the rat AMI model to detect DAPK1 expression changes and used DAPK1 inhibitors to detect their effect on AMI rats and cardiomyocytes.

## 2. Materials and Methods

### 2.1. Animals

80 male Sprague-Dawley (SD) rats (200-250 g) were used in this study. SD rats were housed in the Chengdu First People's Hospital Experimental Animal Center. We use standard sterile rodent feed to feed rats. The rats were in the environment with room temperature of 18-22°C, relative humidity of 45-65% and routine lighting. The experimental environment complies with the standards set by the International Animal Protection Association. The animal experiments in this study have been approved by the Animal Ethics Committee of Chengdu First People's Hospital.

### 2.2. AMI Model

We weighed the rats and used electric clippers to dehair the rats. Then, we used 10 g/L pentobarbital sodium (50 mg/kg) to anesthetize rats by intraperitoneal injection. After the rat lost consciousness, we fixed the rat on the operating table in a supine position. Then, we used an 18G venous indwelling needle for tracheal intubation and connected it to a small animal ventilator (CWE SAR-830, Orange, CA, USA). The small animal ventilator was set to tidal volume of 5-7 mL and frequency of 75 times/min. We used iodophor to disinfect the chest and abdomen of the mouse and then used scissors to cut the skin of the left chest and cut the third and fourth ribs. Then, we used forceps to carefully tear the envelope. Under the cold light spotlight, we carefully identified the left anterior descending coronary artery. The left anterior coronary artery descends to the root of the left atrial appendage. We ligated the coronary arteries with nylon thread and recorded the electrocardiogram of the rat using an electrocardiograph. The elevation of the ST segment of the electrocardiogram indicated that the coronary artery was successfully blocked. We then closed the rat thoracic cavity layer by layer and sutured the surgical incision. The rats were placed on a 37°C water bath. After the rats regained consciousness and mobility, we put the rats back into the rat cage. The rats in the sham group only opened the chest cavity, but the coronary arteries were not ligated. For the treatment group, we used TC-DAPK 6 (1 mg/kg, 2 mg/kg) (MCE, Monmouth Junction, NJ, USA), an inhibitor of DAPK1, to subcutaneously inject AMI rats daily from the first day of modeling. For the nontreatment group, we injected the same amount of normal saline every day.

### 2.3. 2, 3, 5-Triphenyl Tetrazolium Chloride (TTC) Staining

After collecting the rat heart, we used phosphate-buffered saline (PBS) to wash away excess blood from the heart. Then, we put the rat heart in the -20°C refrigerator for refrigeration. After 30 min, we took the rat heart and confirmed whether the heart was completely frozen. Then, we cut the heart into 2 mm slices perpendicular to the longitudinal axis. Heart slices were placed in 2% TTC staining solution (Sigma-Aldrich, St. Louis, MO, USA) and incubated in the dark for 15 min. Normal myocardial tissue appears red, while infarcted myocardium appears pale. We used the percentage of infarcted myocardium to represent the extent of myocardial infarction.

### 2.4. Echocardiography for Detecting Cardiac Function in Rats

1, 3, 7, and 14 d after making the AMI model, we examined the cardiac function of rats by echocardiography. After anesthetizing the rat with 10 g/L pentobarbital sodium (50 mg/kg), we fixed the rat in a supine position and performed cardiac ultrasound using a Vevo high-resolution ultrasound system (VisualSonics, Toronto, Canada). The left ventricular end-systolic diameter and left ventricular end-diastolic diameter were recorded. Left ventricular ejection fraction (LVEF) and left ventricular fractional shortening (LVFS) are automatically calculated by an echocardiogram computer.

### 2.5. Lactic Dehydrogenase (LDH) and Creatine Kinase (CK) Levels in Serum

We collected rat blood and left it at room temperature for 15 min. Then, we obtained the serum by centrifugation (3000 rpm, 15 min, 4°C). LDH activity detection kit (Sigma-Aldrich, St. Louis, MO, USA) and CK activity detection kit (Sigma-Aldrich, St. Louis, MO, USA) were used to detect the level of LDH and CK in serum. We used purified rat LDH antibody coating plate to make solid phase antibody. Then, we added unknown concentration of LDH test sample and known concentration of LDH standards to the monoclonal antibody. After incubating for 15 min, we added biotin-labeled anti-IgG antibody. The IgG antibody then binds to streptavidin-HRP to form an immune complex. Then, we added substrate TMB for color development. The color depth is positively correlated with the LDH in the sample. Finally, we used a microplate reader to measure the absorbance (OD value) at a wavelength of 450 nm and calculated the concentration of rat LDH in the sample through the standard curve. The same principle was also used to detect the level of CK.

### 2.6. Enzyme-Linked Immunosorbent Assay (ELISA)

After obtaining rat serum, we used interleukin- (IL-) 1*β*, IL-6, and tumor necrosis factor- (TNF-) *α* ELISA kits (Sigma-Aldrich, St. Louis, MO, USA) to detect the levels of inflammatory factors in serum. The standards in the ELISA kit were used to make a standard curve. Then, we calculated the concentration of the sample based on the absorbance of the sample and the standard curve.

### 2.7. Activity Detection of Malonaldehyde (MDA), Glutathione (GSH), Superoxide Dismutase (SOD), and Catalase (CAT) in Myocardium and Cell Supernatant

We collected 2 mg of rat myocardial tissue and added 9 times the volumes of normal saline. Then, we used scissors to shred the tissue and add 3 small steel balls to each EP tube. Then, we put the EP tube into the homogenizer for homogenization (10000 r/min) 3 times, 1 min each time. Then, we took the supernatant in the EP tube by centrifugation (12000 rpm, 15 min, 4°C). MDA, GSH, SOD, and CAT activity detection kits (Sigma-Aldrich, St. Louis, MO, USA) were used to detect the levels of MDA, GSH, SOD, and CAT in serum. The detection of MDA, GSH, SOD, and CAT in the cell supernatant was similar to that of myocardial tissue.

### 2.8. Histology and Hematoxylin-Eosin (HE) Staining

After collecting the rat heart, we used PBS to wash away excess blood from the heart. Then, we used 4% paraformaldehyde to fix myocardial tissue for 24 h. The fixed tissue can be stored in PBS for a long time. We put myocardial tissue into gradient alcohol in order to dehydrate. Then, we put myocardial tissue in xylene and paraffin solution to make paraffin blocks. The microtome is used to make paraffin sections with the thickness of 5 *μ*m. We put paraffin sections in a 37°C incubator for 3 d. Then, we put the paraffin sections in xylene solution and gradient alcohol sequentially. After washing the sections with running water, we stained the cell nucleus with hematoxylin stain (Beyotime, Shanghai, China). Then, we put the sections in 1% hydrochloric acid alcohol for 3 s and quickly rinsed the sections with running water. Then, we put the sections in eosin stain (Beyotime, Shanghai, China) and gradient alcohol in turn for 3 min each time. Finally, we use neutral gum for mounting and observe the staining results using an optical microscope.

### 2.9. Immunohistochemical (IHC) Staining

After dewaxing and hydration using xylene and gradient alcohol, we rinsed the sections in running water for 3 min. We put the sections in citrate buffer. The buffer just barely goes through the sections. Then, we heated the citrate buffer slowly to 95°C in a water bath for 20 min. After it naturally cooled to room temperature, we took out the sections and washed it with PBS three times for 3 min each time. Then, we put the sections in a wet box and incubate them for 30 min with 3% hydrogen peroxide on myocardial tissue. Then, we blocked the nonspecific antigen with 10% goat serum and incubated them at 4°C overnight with primary antibody dilution (DAPK1, ab200549; peroxiredoxin (Prdx)1, ab15571; Prdx4, ab59542; Abcam, Cambridge, MA, USA). After washing the sections with PBS, we incubated them with secondary antibody dilution (GeneTech, Shanghai, China) for 1 h and used DAB for color development. Hematoxylin is used in the cell nucleus. Finally, we used an optical microscope to observe the results.

### 2.10. Cell Culture

The rat cardiomyocyte cell line, H9c2 cells, was used in this study. DMEM medium containing 10% fetal bovine serum (FBS) (Gibco, Rockville, MD, USA) and 1% double antibody (Gibco, Rockville, MD, USA) was used to culture H9c2 cells. IL-1*β* (100 ng/mL) (Invitrogen, Carlsbad, CA, USA) was used to stimulate cell injury.

### 2.11. RNA Isolation and Quantitative Real-Time Reverse Transcription-Polymerase Chain Reaction (RT-PCR)

The TRIzol (Sigma-Aldrich, St. Louis, MO, USA) method was used to extract total RNA from myocardial tissue and H9c2 cells. Then, we used a spectrophotometer to detect RNA concentration and A260/A280. Samples with A260/A280 around 2.0 can be used for subsequent experiments. We took 0.5 *μ*g total RNA (5 *μ*L) for reverse transcription. The 10 *μ*L reverse transcription system is 5 + 3 *μ*L RNase-free H_2_O+2 *μ*L 5x HiScript II Q RT SuperMix (Vazyme, Nanjing, Jiangsu, China). The PCR instrument was set to 50°C for 15 min, 85°C for 5 s, and 4°C for 30 min. SYBR Green Master Mix (Vazyme, Nanjing, Jiangsu, China) was used for real-time RT-PCR. The reaction system is 5 *μ*L SYBR Green Master Mix +0.4 *μ*L Primer forward/reverse +1 *μ*L cDNA template +3.6 *μ*L ddH_2_O. Reaction temperature and time refer to primer company's instructions. The endogenous GAPDH expression was used as a reference. The relative expression of RNA is expressed as 2^−ΔΔCt^. The primer sequences are shown in Table 1.

### 2.12. Flow Cytometry Analyses

Flow cytometry was used to detect reactive oxygen species (ROS) levels. Pancreatin was used to prepare single-cell suspensions. Then, we took 1 mL of single-cell suspension and added 5 *μ*L of 2′, 7′-dichlorodihydrofluorescein diethyl ester (DCF-DA) (Sigma-Aldrich, St. Louis, MO, USA) for 30 min. Then, we put it in a centrifuge (1500 rpm, 5 min) and discard the supernatant. Then, we added 10% fetal bovine serum and incubated it at 37°C for 20 min. After centrifuging it again in a centrifuge (1500 rpm, 5 min), we discarded the supernatant and added cold PBS. Then, we used flow cytometry (Thermo Fisher Scientific, Waltham, MA, USA) to detect the average fluorescence intensity of the fluorescent probes in the cells. The relative fluorescence intensity value is the ratio of the fluorescence intensity value of the experimental group to that of the control group.

### 2.13. Cell Counting Kit-8 (CCK8)

CCK8 (Dojindo Molecular Technologies, Kumamoto, Japan) was used to detect the proliferation ability of H9c2 cells. We used 96-well plates to culture H9c2 cells. 10 *μ*L of CCK8 reagent was added to each well of a 96-well plate. The 96-well plate was placed in a 37°C incubator for 2 h in the dark. Then, we used a microplate reader to detect the absorbance (OD value) of each well at 450 nm. There was only the medium and no cells in the blank wells, and there were medium and noninterfering cells in control wells. Cell viability = (OD intervention − OD blank)/(OD control − OD blank).

### 2.14. Statistical Analysis

Statistical Product and Service Solutions (SPSS) 20.0 statistical software (IBM, Armonk, NY, USA) was used for the statistical analysis of this study. Graphpad prism 7.0 software (La Jolla, CA, USA) was used for graphing in this study. We use the mean ± standard deviation to represent the study data. Differences between two groups were analyzed by using Student's *t*-test. Comparison between multiple groups was done using one-way ANOVA test followed by post hoc test (least significant difference). *P* < 0.05 was considered statistically significant.

## 3. Results

### 3.1. High Expression of DAPK1 in Myocardium of AMI Rats

**Figure 1:**
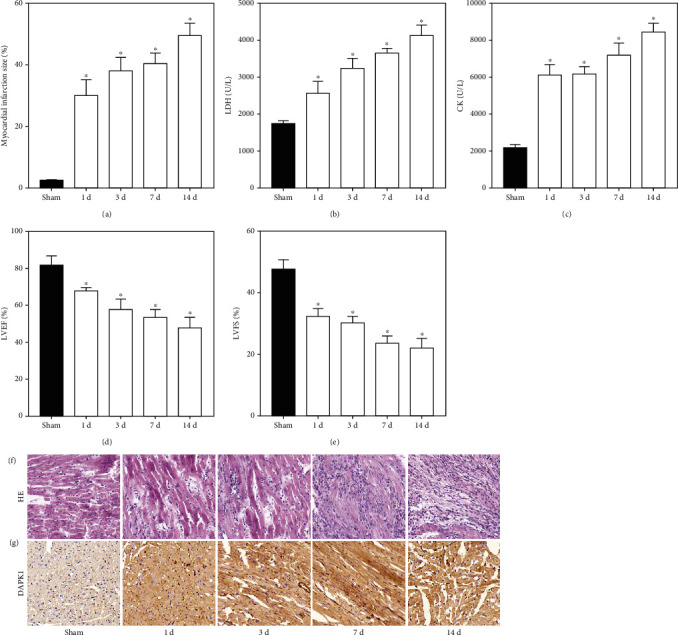
High expression of DAPK1 in myocardium of AMI rats. After 1, 3, 7, and 14 d of coronary artery occlusion, the myocardial infarction size (a), myocardial injury markers (b, c), and cardiac function (d, e) were detected to confirm modeling efficiency. HE staining (f) also demonstrated the destruction of myocardial structure due to coronary artery occlusion (magnification: 200x). IHC staining (g) showed that DAPK1 expression increased in AMI rats (magnification: 200x) (“^∗^” means *P* < 0.05 vs. sham group).

After 1, 3, 7, and 14 d of blocking the rat coronary arteries, we took rat myocardial tissue for TTC staining (Figure 1(a)). We found that the scope of myocardial infarction gradually increased with time. The concentrations of myocardial infarction markers LDH (Figure 1(b)) and CK (Figure 1(c)) also increased with the time of myocardial infarction. In addition, we detected the cardiac function of rats by echocardiography. The LVEF (Figure 1(d)) and LVFS (Figure 1(e)) of AMI rats were significantly lower than that of the sham group and decreased with time. HE staining (Figure 1(f)) detected the structure of rat myocardial tissue. The arrangement of myocardial cells in AMI rats was disordered, and the morphology of myocardial cells also changed. The myocardial injury in AMI rats was most obvious after 14 d and was accompanied by the infiltration of inflammatory cells. IHC staining (Figure 1(g)) detected the expression of DAPK1 in rat myocardium. We found that with the increase of myocardial infarction time, the expression of DAPK1 in myocardial tissue gradually increased.

### 3.2. Inhibition of DAPK1 Alleviates Cardiac Dysfunction and Inflammation in AMI Rats

**Figure 2:**
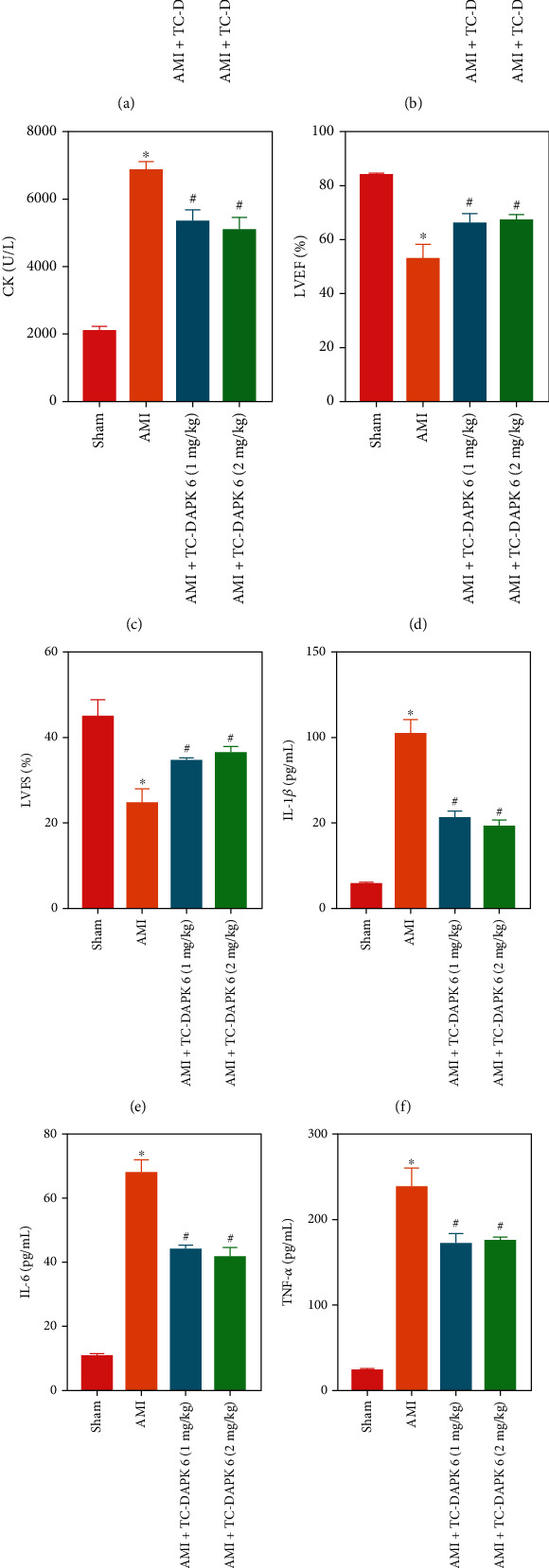
Inhibition of DAPK1 alleviates cardiac dysfunction and inflammation in AMI rats. TC-DAPK 6 was used to treat AMI rats and to detect changes in myocardial infarction size (a), myocardial injury markers (b, c), and cardiac function (d, e) in AMI rats. The expression of inflammatory cytokines (IL-1*β*, IL-6, and TNF-*α*) in rat serum was detected by ELISA (f–h). HE staining (i) was used to detect the structural changes of rat myocardial tissue (magnification: 200x) (“^∗^” means *P* < 0.05 vs. sham group, and “^#^” means *P* < 0.05 vs. AMI group).

Considering the myocardial injury of the 14 d AMI rats was too severe, we chose 7 d AMI rats for subsequent experiment. We divided the rats into sham, AMI, AMI + TC-DAPK 6 (1 mg/kg), and AMI + TC-DAPK 6 (2 mg/kg) groups. The results of TTC staining (Figure 2(a)) showed that after inhibition of DAPK1, the range of myocardial infarction in rats was reduced. Myocardial infarction markers LDH (Figure 2(b)) and CK (Figure 2(c)) also decreased with the inhibition of DAPK1. The inhibition of DAPK1 also improved the cardiac function of rats, which was manifested by the increase of LVEF (Figure 2(d)) and LVFS (Figure 2(e)). In addition, we detected changes in inflammatory factors by ELISA. After TC-DAPK 6 was used to treat AMI rats, the expressions of IL-1*β* (Figure 2(f)), IL-6 (Figure 2(g)), and TNF-*α* (Figure 2(h)) in rat serum were all reduced. HE staining (Figure 2(i)) results also prove that TC-DAPK 6 improved the structure of myocardial tissue. However, there was no significant difference between the AMI + TC-DAPK 6 (1 mg/kg) and AMI + TC-DAPK 6 (2 mg/kg) groups.

### 3.3. Inhibition of DAPK1 Alleviates AMI-Induced Oxidative Injury

**Figure 3:**
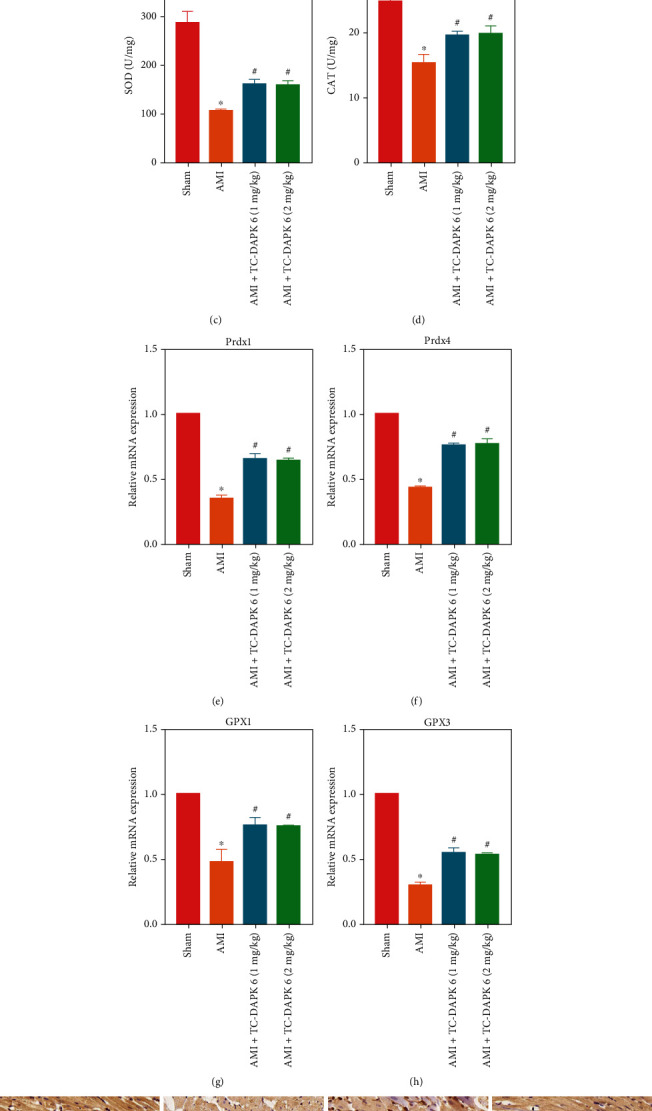
Inhibition of DAPK1 alleviates AMI-induced oxidative injury. The activities of MDA (a), GSH (b), SOD (c), and CAT (d) were measured to determine the level of oxidative stress in rat myocardial tissue. The mRNA expression of Prdx1 (e), Prdx4 (f), GPX1 (g), and GPX3 (h) was detected by RT-PCR. The protein expression of Prdx1 and Prdx4 was detected by IHC staining (magnification: 200x) (i) (“^∗^” means *P* < 0.05 vs. sham group, and “^#^” means *P* < 0.05 vs. AMI group).

The effect of DAPK1 on oxidative stress of myocardial tissue has also been studied. We detected the activities of MDA (Figure 3(a)), GSH (Figure 3(b)), SOD (Figure 3(c)), and CAT (Figure 3(d)) in rat myocardium. MDA activity of AMI rats was significantly higher than that of sham group, while GSH, SOD, and CAT activities of AMI group were lower than that of sham group. Inhibition of DAPK1 was found to attenuate the AMI-induced changes. RT-PCR detected the expression of Prdx1 (Figure 3(e)), Prdx4 (Figure 3(f)), GPX1 (Figure 3(g)), and GPX3 (Figure 3(h)). Their expression decreased in myocardial tissue of AMI rats, and the inhibition of DAPK1 increased the expression of these indicators. IHC staining (Figure 3(i)) also proved the promoting effect of TC-DAPK 6 on Prdx1 and Prdx4. However, there was no significant difference between the AMI + TC-DAPK 6 (1 mg/kg) and AMI + TC-DAPK 6 (2 mg/kg) groups.

### 3.4. Inhibition of DAPK1 Alleviates IL-1*β*-Induced H9c2 Cell Injury

**Figure 4:**
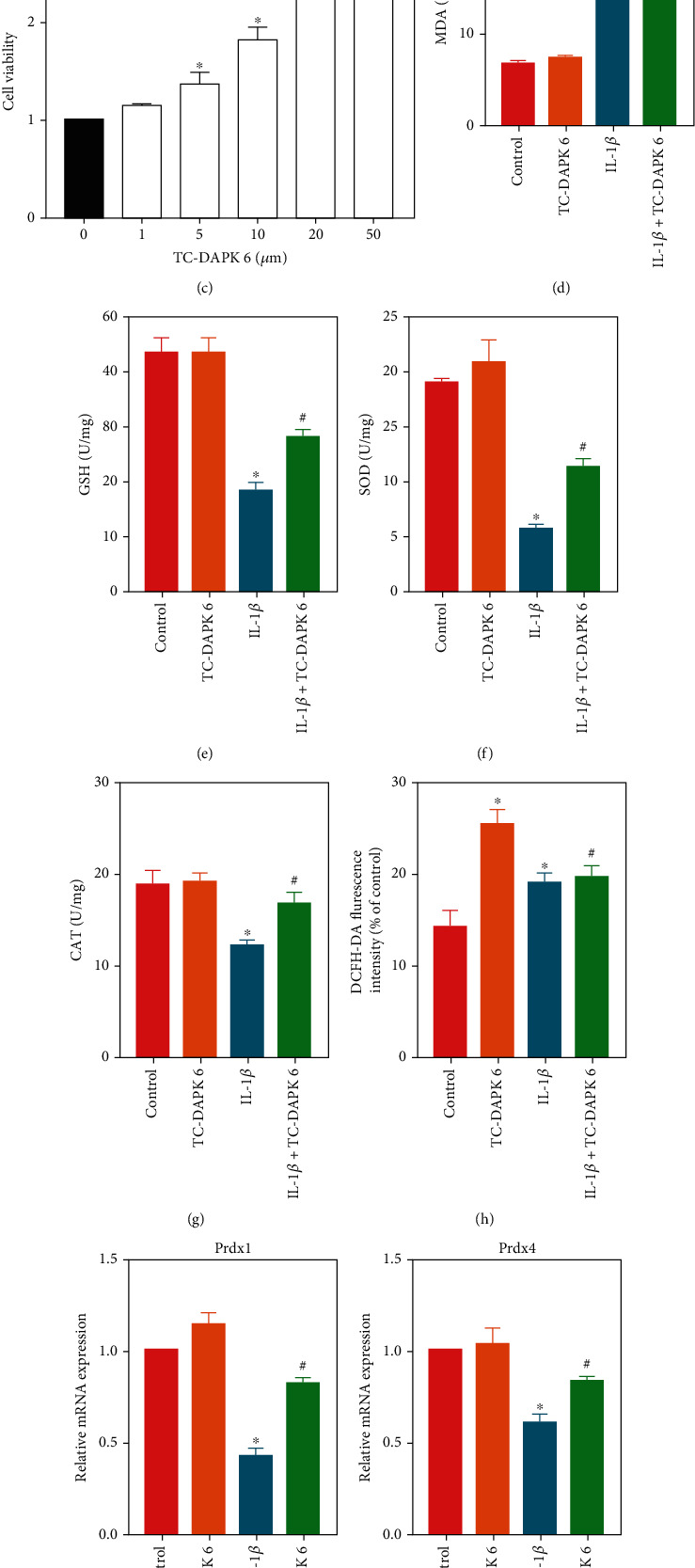
Inhibition of DAPK1 alleviates IL-1*β*-induced H9c2 cell injury. Expression of DAPK1 in H9c2 cells was detected by IF staining (magnification: 200x) (a) and RT-PCR (b). CCK8 (c) detected the effect of 1, 5, 10, 20, and 50 *μ*M TC DAPK 6 on H9c2 cells viability. The activities of MDA (d), GSH (e), SOD (f), and CAT (g) were measured to determine the level of oxidative stress in H9c2 cells. ROS level in H9c2 cells was detected by flow cytometry (h). The mRNA expression of Prdx1 (i), Prdx4 (j), GPX1 (k), and GPX3 (l) was detected by RT-PCR (“^∗^” means *P* < 0.05 vs. control group, and “^#^” means *P* < 0.05 vs. IL-1*β* group).

We verified the effect of DAPK1 on cardiomyocytes in H9c2 cells. IL-1*β* is used to induce H9c2 cell injury. We found by IF staining (Figure 4(a)) and RT-PCR (Figure 4(b)) that after IL-1*β* stimulated H9c2 cells for 1 d and 3 d, the expression of DAPK1 mRNA and protein gradually increased. We used 1, 5, 10, 20, and 50 *μ*M TC-DAPK 6 to stimulate H9c2 cells and detect cell viability by CCK8 (Figure 4(c)). 20 *μ*M was found to be the optimal concentration for TC-DAPK 6 to stimulate H9c2 cells, so we used 20 *μ*M TC-DAPK 6 for subsequent experiments. We divided H9c2 cells into control, TC-DAPK 6, IL-1*β*, and IL-1*β* + TC-DAPK 6 groups. The activities of MDA (Figure 4(d)), GSH (Figure 4(e)), SOD (Figure 4(f)), and CAT (Figure 4(g)) were detected, and we found that IL-1*β* increased the level of oxidative stress, while TC-DAPK 6 can attenuate oxidative stress injury induced by IL-1*β*. Flow cytometry (Figure 4(h)) detected ROS levels, and the results showed that TC-DAPK 6 can also reduce ROS levels in H9c2 cells. RT-PCR results also indicated that TC-DAPK 6 can promote the expression of antioxidant molecules Prdx1 (Figure 4(i)), Prdx4 (Figure 4(j)), GPX1 (Figure 4(k)), and GPX3 (Figure 4(l)).

## 4. Discussion

With the formation of fibrous scar tissue and ventricular remodeling after AMI, myocardial contractility gradually decreases and eventually leads to heart failure and even death [9]. At present, DAPK1 is expected to become a new target for AMI treatment. We found high expression of DAPK1 in AMI rats. The use of DAPK1 inhibitors significantly improved the cardiac function and myocardial injury in rats and alleviated the H9c2 cell injury induced by IL-1*β*. Therefore, DAPK1 inhibitors are expected to become new drugs for clinical treatment of AMI.

Coronary artery occlusion is the main cause of AMI, and the most intuitive manifestation of AMI is the increase in the extent of myocardial infarction [10]. A study investigated the effect of melatonin receptor agonist, ramelteon, on myocardial protection through the model of myocardial ischemia-reperfusion. They found that after coronary artery occlusion and recanalization, the infarct size of the rat heart increased significantly [11]. We made the AMI model by occluding coronary arteries and observed the changes of myocardial infarction size in rats by TTC staining. LDH and CK are important indicators for judging myocardial injury, and their expression in normal myocardial tissue is low. When the body is subjected to a stress response or cardiac ischemia, LDH and CK in the blood increase significantly [12]. Therefore, in order to determine the success rate of modeling, we also detected the activity of LDH and CK in the serum of AMI rats. The range of myocardial infarction, the activity of LDH and CK, and the detection of rat cardiac function proved that the AMI rat model was successfully made. However, considering the myocardial injury of the 14-day-old model rats was very serious, we chose 7 d as the optimal time for modeling, in order to prevent obscuring the therapeutic effect of the intervention.

The inflammation triggered after AMI plays a vital role in determining the extent of myocardial infarction and subsequent ventricular remodeling [13, 14]. After the occurrence of AMI, a large number of myocardial cells rupture due to ischemia, hypoxia, and necrosis, inducing the activation of nonspecific immune responses [15, 16]. Therefore, a large number of inflammatory cytokines are released and enter the blood circulation, such as IL-6, IL-1*β*, and TNF-*α*. The inflammatory factors promote the activation of inflammatory cells such as neutrophils, macrophages, and natural killer cells. These cells are chemoattracted to damaged myocardium and participate in tissue repair and ventricular remodeling [17]. A study on the relationship among inflammation, autophagy, and apoptosis in myocardial infarction found that inflammation, autophagy, and apoptosis have a common role in the course of myocardial infarction [18]. In addition, data from a clinical study also revealed that inflammation affects the hospitalization and prognosis of elderly AMI patients [19]. We also found similar results in this study. Inflammatory factors (IL-1*β*, IL-6, and TNF-*α*) in serum of AMI rats were significantly increased, but the application of DAPK1 inhibitors reduced the myocardial inflammation.

When AMI occurs, it will destroy the balance of the oxidative and antioxidative systems in the myocardial tissue, resulting in the generation of large amounts of oxygen free radicals. At the same time, the activity of SOD and GSH will decrease, leading to the accumulation of oxygen free radicals in the body [20]. Oxygen free radicals can act on lipids to peroxidation to produce MDA, causing the cross-linking polymerization of proteins, nucleic acids, and other life macromolecules, so oxygen free radicals are also cytotoxic [21]. The sum of the results of four large cohort studies from Europe found that oxidative stress injury has a clear association with AMI. They found that the imbalance of the redox system was involved in the pathogenesis of AMI [22]. In another study, oxidative stress injury was restricted by interfering with the Akt signaling pathway in cardiomyocytes; therefore, cardiomyocyte death caused by AMI was reduced [23]. Wang et al. found a decrease in SOD and GSH and an increase in MDA in a mouse model of septic heart dysfunction, suggesting a decrease in the antioxidant capacity of sepsis mice, while dexmedetomidine can reverse these changes and alleviate heart dysfunction in mice [24]. Therefore, we also detected the oxidative stress level of rat myocardial tissue and H9c2 cells. The decrease of MDA activity and the increase of GSH, SOD, and CAT activities suggested the antioxidation effect of DAPK1 inhibitors. Prdx1/4 and GPX1/3 are important antioxidant molecules, and the inhibition of DAPK1 also promotes their expression in cardiomyocytes.

Therefore, DAPK1 plays an important role in the occurrence and development of AMI. The DAPK1 inhibitors may have potential application prospects for the treatment of AMI. We hope that this study can provide new targets for clinical treatment of AMI.

## 5. Conclusions

DAPK1 was highly expressed in the myocardium of AMI rats and affects the occurrence and development of AMI. DAPK1 inhibitors reduce the levels of inflammation and oxidative stress in myocardial tissues and cells, thereby improving the structure and function of the rat heart. Therefore, DAPK1 inhibitors have potential application prospects for the treatment of AMI.

## Figures and Tables

**Table 1:** Primer sequences for RT-PCR.

Name	Sense/antisense	Sequences (5′-3′)
DAPK1	Sense	CACCTCACTCCCTTCCC
	Antisense	TCACCCACAGACGGATG
Prdx1	Sense	TTCTGTCATCTGGCATGG
	Antisense	CCCAATCCTCCTTGTTTCT
Prdx4	Sense	CCAGCACCTTATTGGGAA
	Antisense	GCGATGATTTCAGTTGGAC
GPX1	Sense	TTGAGAAGTGCGAGGTGAA
	Antisense	TCCGCAGGAAGGTAAAGAG
GPX3	Sense	CGGGGAGAAAGAGCAGAA
	Antisense	CAAAGTTCCAGCGGATGTC
GAPDH	Sense	ATGGCTACAGCAACAGGGT
	Antisense	TTATGGGGTCTGGGATGG

## Data Availability

The datasets used and analyzed during the current study are available from the corresponding author on reasonable request.
